# MDL28170, a Calpain Inhibitor, Affects *Trypanosoma cruzi* Metacyclogenesis, Ultrastructure and Attachment to *Rhodnius prolixus* Midgut

**DOI:** 10.1371/journal.pone.0018371

**Published:** 2011-04-04

**Authors:** Vítor Ennes-Vidal, Rubem F. S. Menna-Barreto, André L. S. Santos, Marta H. Branquinha, Claudia M. d'Avila-Levy

**Affiliations:** 1 Laboratório de Biologia Molecular e Doenças Endêmicas, Instituto Oswaldo Cruz (IOC), Fundação Oswaldo Cruz (Fiocruz), Rio de Janeiro, Brazil; 2 Laboratório de Biologia Celular, IOC, Fiocruz, Rio de Janeiro, Brazil; 3 Laboratório de Estudos Integrados em Bioquímica Microbiana, Departamento de Microbiologia Geral, Instituto de Microbiologia Prof. Paulo de Góes (IMPPG), Universidade Federal do Rio de Janeiro (UFRJ), Rio de Janeiro, Brazil; 4 Laboratório de Bioquímica de Proteases, Departamento de Microbiologia Geral, IMPPG, UFRJ, Rio de Janeiro, Brazil; Universidade Federal do Rio de Janeiro, Brazil

## Abstract

**Background:**

*Trypanosoma cruzi* is the etiological agent of Chagas' disease. During the parasite life cycle, many molecules are involved in the differentiation process and infectivity. Peptidases are relevant for crucial steps of *T. cruzi* life cycle; as such, it is conceivable that they may participate in the metacyclogenesis and interaction with the invertebrate host.

**Methodology/Principal Findings:**

In this paper, we have investigated the effect of the calpain inhibitor MDL28170 on the attachment of *T. cruzi* epimastigotes to the luminal midgut surface of *Rhodnius prolixus*, as well as on the metacyclogenesis process and ultrastructure. MDL28170 treatment was capable of significantly reducing the number of bound epimastigotes to the luminal surface midgut of the insect. Once the cross-reactivity of the anti-*Dm*-calpain was assessed, it was possible to block calpain molecules by the antibody, leading to a significant reduction in the capacity of adhesion to the insect guts by *T. cruzi*. However, the antibodies were unable to interfere in metacyclogenesis, which was impaired by the calpain inhibitor presenting a significant reduction in the number of metacyclic trypomastigotes. The calpain inhibitor also promoted a direct effect against bloodstream trypomastigotes. Ultrastructural analysis of epimastigotes treated with the calpain inhibitor revealed disorganization in the reservosomes, Golgi and plasma membrane disruption.

**Conclusions/Significance:**

The presence of calpain and calpain-like molecules in a wide range of organisms suggests that these proteins could be necessary for basic cellular functions. Herein, we demonstrated the effects of MDL28170 in crucial steps of the *T. cruzi* life cycle, such as attachment to the insect midgut and metacyclogenesis, as well as in parasite viability and morphology. Together with our previous findings, these results help to shed some light on the functions of *T. cruzi* calpains. Considering the potential roles of these molecules on the interaction with both invertebrate and vertebrate hosts, it is interesting to improve knowledge on these molecules in *T. cruzi*.

## Introduction

Chagas' disease is a neglected tropical disease, which remains a major health problem in Latin America. Over eight million people are infected with this disease and an estimated of 14,000 people die as a consequence of the infection every year [1]. The etiological agent *Trypanosoma cruzi* undergoes profound morphological changes during its development in a complex life cycle involving mammalian and invertebrate hosts. The protozoa life cycle comprises three major morphological stages: epimastigotes, trypomastigotes, and amastigotes [2]. During the infection of the invertebrate host, a hemipteran insect of the Reduviidae order, non-infectious epimastigotes adhere to the insect host midgut, begin to proliferate and differentiate (metacyclogenesis process) into metacyclic trypomastigotes, which are non-proliferative forms that are able to infect a mammalian host [3]. The adhesion to the luminal midgut surface of the insect appears to be necessary for the metacyclogenesis, but there is a general lack of information about which molecules are implicated in this process [3], [4]. In this context, peptidases, a class of hydrolytic enzymes responsible for breaking peptide bonds, has attracted the attention of our research group because of their role in several crucial steps of the life cycle of the trypanosomatid parasites [5]. Among *T. cruzi* different peptidases that we considered, the calpains have been presenting interesting findings and seem to be a remarkable target for the development of an alternative target to treat Chagas' disease and leishmaniasis [6], [7], [8].

Calpains constitute a large family of calcium-regulated cytosolic cysteine peptidases that have been characterized mainly in humans and whose role still remains poorly understood [9]. Some evidence indicates that these enzymes may participate in a variety of cellular processes, including the rearrangement of cytoskeletal proteins, different signal transduction pathways and apoptosis. In this context, a variety of calpain inhibitors are under development and the potential clinical utility of these compounds have been shown mainly in the treatment of neurodegenerative disorders [10], [11], [12], [13]. In this sense, a classical study employing whole genome analyses showed the presence of a large and diverse family of calpains in *Trypanosoma brucei*, *Leishmania major* and *T. cruzi* [14]. Some years before, the same group had already characterized a trypanosomatid calpain-like protein in procyclic forms of *T. brucei* [15]. In addition, our group described the presence of calpain-related proteins in *T. cruzi* epimastigote forms and *Leishmania amazonensis* promastigote forms and the effects of the calpain inhibitor III (MDL28170) on growth, viability and infectivity [6], [7], [8]. Calpain homologues were also described in the monoxenic trypanosomatids *Crithidia deanei* and *Herpetomonas samulpessoai* [16], [17].

More studies are necessary to better understand the involvement of the calpain homologues in the life cycle of *T. cruzi*. Here, we have conducted a study to investigate the effect of the calpain inhibitor MDL28170 on the attachment of *T. cruzi* epimastigotes to the luminal midgut surface of *Rhodnius prolixus*, as well as on the metacyclogenesis process and ultrastructure. In addition, we have analyzed the effect of anti-calpain antibodies on the interaction of epimastigote forms to the midgut surface of the insect and on the metacyclogenesis.

## Methods

### Ethics Statement

The experiments were carried out in accordance with the guidelines established by the FIOCRUZ Committee of Ethics for the Use of Animals (CEUA L-028/09).

### Chemicals

The calpain inhibitor III, MDL28170 (carbobenzoxy-valylphenylalanial; Z-Val-Phe-CHO), was purchased from Calbiochem (San Diego, CA, USA). Stock solutions of the drug (5 mM) were prepared in dimethylsulfoxide (DMSO). All other reagents were analytical grade or superior.

### Parasite culture

Epimastigote forms of *T. cruzi* were grown in 3.7% brain heart infusion medium (BHI), containing hemin and folic acid and supplemented with 10% heat-inactivated fetal bovine serum, at 28°C for 4 days to reach late-log phase growth. For the following experiments, epimastigotes were collected, washed three times in 0.15 M NaCl, 0.01 M phosphate-buffer pH 7.2 (PBS) and immediately used. The Y strain of *T. cruzi* was used in all experiments except for the metacyclogenesis assay, in which the Dm28c strain is the best characterized model for in vitro differentiation [18].

### Insects

*Rhodnius prolixus* were reared and maintained as previously described [19]. Briefly, fifth-instars larvae were starved for 30 days after the last ecdysis and then allowed to feed on rabbit blood through a membrane feeder. Ten days after the feeding, insects were dissected; the posterior midguts were then removed, longitudinally sectioned and washed three times in PBS to expose their luminal surfaces. After the washing, the tissue fragments were processed as described below. The insects were obtained from the insectary of the Laboratório Nacional e Internacional de Referência em Taxonomia de Triatomíneos, Instituto Oswaldo Cruz, FIOCRUZ.

### Identification of calpain homologues by flow cytometry and fluorescence microscopy

Epimastigotes (1×10^7^ cells) from the Y strain used for these experiments were fixed at 4°C in 0.4% paraformaldehyde in PBS (pH 7.2) for 30 min, followed by extensive washing in the same buffer. The fixed cells maintained their morphological integrity, as verified by optical microscopic observation. After this step, the cells were incubated for 1 h at room temperature with a 1∶100 dilution of the anti-calpain antibodies. Cells were then incubated for an additional hour with a 1∶200 dilution of fluorescein isothiocyanate (FITC)-labeled goat anti-rabbit IgG [20]. The cells were then washed 3 times in PBS and observed in a Zeiss epifluorescence microscope (Axioplan 2). Alternatively, the parasite associated fluorescence was excited at 488 nm and quantified on a flow cytometer (FACSCalibur, BD Bioscience, USA) equipped with a 15 mW argon laser emitting at 488 nm. Non-treated cells and those treated with the secondary antibody alone were run in parallel as controls. Each experimental population was then mapped by using a two-parameter histogram of forward-angle light scatter versus side scatter. The mapped population (n = 10,000) was then analyzed for log green fluorescence by using a single parameter histogram. The calpain antibodies tested were: a rabbit antiserum raised against *Drosophila melanogaster* calpain (anti-*Dm*-calpain) [21], anti-C21, anti-C23 or anti-C24 raised against the whole molecule, the cysteine active site and the histidine active site, respectively, of human brain m-calpain [22], anti-CAP5.5, raised against the cytoskeleton-associated protein from *Trypanosoma brucei* [15], and anti-CDPIIb and anti-*Ha*-CalpM raised against *Homarus americanus* calpains [23], [24].

### *In vitro* inhibition of *T. cruzi-Rhodnius* interaction

Live epimastigote forms (Y strain) were resuspended in 200 µl of fresh BHI to a density of 1×10^7^ cells and treated with the calpain inhibitor for 1 h with sub-inhibitory concentrations (6.25–50 µM) and washed in PBS. Under this experimental condition, the parasite maintains their viability, as previously described [7]. Dilutions of DMSO corresponding to those used to prepare the drug solution were assessed in parallel for control. Alternatively, the parasites were incubated for 1 h with anti-calpain antibodies, or rabbit pre-immune sera. After that, binding of protozoa to insect gut was performed by a method similar to that described previously [25]. Briefly, the parasites were incubated for 20 min at 28°C with *R. prolixus* dissected posterior midguts that were sliced open longitudinally. Subsequently, the explanted midguts were spread onto glass slides to count the number of attached parasites per epithelial cells. The experiment was performed three times independently, for each experimental group 4 insect midguts were used and 100 epithelial cells were counted randomly.

### *In vitro* inhibition of *T. cruzi* metacyclogenesis

For *in vitro* differentiation, epimastigotes (clone Dm28c) in the stationary phase of growth were incubated for 2 h at 28°C in triatomine artificial urine (TAU) medium (190 mM NaCl, 17 mM KCl, 2 mM MgCl_2_, 2 mM CaCl_2_, 8 mM phosphate buffer pH 6.0) at a density of 5×10^8^ cells/ml. The parasites at a dilution of 1∶100 were further incubated for 96 h in TAU3AAG medium (TAU supplemented with 10 mM L-proline, 50 mM L-sodium glutamate, 2 mM L-sodium aspartate, and 10 mM D-glucose) in culture flasks [18]. For inhibition assays, epimastigotes were incubated in the presence, or absence (control), of increasing concentrations of MDL28170 (6.25–50 µM) in TAU3AAG medium or anti-calpain antibodies. Culture supernatants were collected after 24, 48, 72, and 96 h of incubation in TAU3AAG medium and the number of epimastigotes and metacyclic trypomastigotes was determined by cell counting in a Neubauer chamber. Under this experimental condition, the parasite maintains their viability, as previously described [7]. These morphological stages can be easily differentiated on morphological grounds by light microscopy, since epimastigotes are broader and have a quite rigid body, while trypomastigotes are slimmer and present a vigorous wavy movement of the whole body. Three independent experiments were performed in triplicate and a DMSO dilution corresponding to highest drug concentration was assessed in parallel.

### Effects of MDL28170 on *T. cruzi* trypomastigotes viability

Bloodstream trypomastigote forms, obtained from infected albino Swiss mice at the peak of parasitemia by differential centrifugation, were resuspended in Dulbecco's modified Eagle medium supplemented with 10% fetal calf serum (DMEM). This suspension was incubated at 37°C for 24 h in the presence of increasing concentrations of MDL28170 (6.25–50 µM). Viability was assessed by mobility of the parasite flagellum and lack of staining after challenge with trypan blue. Dilutions of DMSO corresponding to those used to prepare the drug solutions were assessed in parallel. Thereafter, the number of viable motile trypomastigotes was quantified by counting the flagellates in a Neubauer chamber.

### Transmission electron microscopy

Briefly, epimastigotes from the Y strain (5×10^6^ cells/ml) were treated with the 50% inhibitory concentration (IC_50_) of MDL28170, as previously determined [7] for 72 h in BHI medium at 28°C. Afterwards, the parasites were fixed with 2.5% glutaraldehyde in 0.1 M Na-cacodylate buffer (pH 7.2) at room temperature for 40 min at 25°C and post-fixed with a solution of 1% OsO_4_, 0.8% potassium ferricyanide and 2.5 mM CaCl_2_ in the same buffer for 20 min at 25°C [26]. The cells were dehydrated in an ascending acetone series and embedded in PolyBed 812 resin. Ultrathin sections were stained with uranyl acetate and lead citrate and examined in Jeol JEM1011 transmission electron microscope. Alternatively, untreated epimastigotes were submitted to pre-embedding protocol, whereas the parasites were fixed, permeabilized (Triton X-100 0.1%), and incubated with polyclonal rabbit anti-*Dm*-calpain (dilution 1∶10), followed by labeling with the secondary anti-rabbit-gold (10 nm) antibody (dilution 1∶10) before the routine protocol described above.

### Statistical analysis

All experiments were repeated at least three times, and media or representative images of these experiments are shown. The data were analyzed statistically using Student's *t* test using EPI-INFO 6.04 (Database and Statistics Program for Public Health) computer software. *P* values of 0.05 or less were considered statistically significant.

## Results

**Figure 1:**
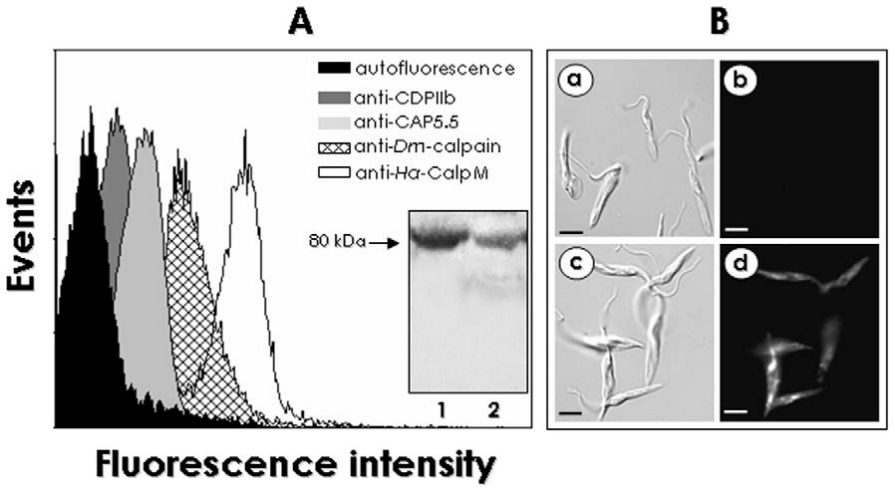
Detection of cross-reactivity between a calpain-like protein from *Trypanosoma cruzi* Y strain and anti-calpain antibodies. (**A**) Flow cytometric analysis showing the anti-calpain antibodies binding to *T. cruzi* epimastigote forms. Paraformaldehyde-fixed cells were incubated in the absence (autofluorescence) or in the presence of 4 anti-calpain antibodies: anti-*Dm*-calpain, anti-CDPIIb, anti-*Ha*-CalpM and anti-CAP5.5 (1∶100 dilution) and analyzed by flow cytometry. When treated only with the secondary-FITC antibody, cells generated similar curves to that observed in the autofluorescence of cells (data not shown). Representative data of the analysis of 10000 cells from 1 of 3 experiments are shown. The inset shows the Western blotting analysis of polypeptides from *T. cruzi* (lane 1) and *D. melanogaster* cell extract (lane 2) probed with anti-*Dm*-calpain antibody. (**B**) Fluorescence microscopy showing the labeling of *T. cruzi* with the anti-*Dm*-calpain antibody. Fixed cells were analyzed under differential interferential contrast images (a, c) and immunofluorescence (b, d). Parasites treated only with the secondary antibody presented no fluorescence intensity (b). The bars represent 1 µm.

The presence of calpain-like molecules in epimastigote forms of *T. cruzi* Y strain was accessed by flow cytometry assay and fluorescence microscopy analyzes using a panel of antibodies raised against different calpains. The antibodies anti-*Dm*-Calpain, anti-CDPIIb, anti-*Ha*-CalpM and anti-CAP5.5 were capable of strongly binding to *T. cruzi* cells, while anti-C21, anti-C23 and anti-C24 did not recognize epitopes on the parasite cell surface (Fig. 1A). However, no cross-reactive of the antibodies with trypomastigote forms were observed. Fluorescence microscopy (Fig. 1B) and FACS (Fig. 1A) with non-permeabilized parasites are suggestive of a surface distribution of calpain-related molecules in epimastigote forms of *T. cruzi*. It has been previously shown by our research group that the anti-*Dm*-Calpain recognizes a polypeptide band migrating at approximately 80 kDa in cellular extracts from the Dm28c strain [7]. Here, we showed that this antibody also recognized a protein with a similar molecular mass (80 kDa) in cellular extracts of *T. cruzi* Y strain (Fig. 1A, inset). For control, lysates of *Drosophila melanogaster* were run in parallel, revealing a reactive band in the same molecular range (Fig. 1A, inset). As previously reported, the fragment of the *D. melanogaster* protein CAA55297.1 that was employed to generate the antibody used in the present report [21] was compared in a BlastP analysis with *T. cruzi* proteins found in GenBank data base. The first 15 hits (homologues with e-value ranging from 2e-8 to 0.003) all corresponded to calcium-dependent cysteine peptidases and had their theoretical molecular mass determined, and 4 out of these 15 homologues presented a molecular mass around 80 kDa: XP_816697.1 (78.3 kDa), XP_803757.1 (80.8 kDa), XP_820102.1 (82.4 kDa) and XP_816696.1 (82.6 kDa), which supports the recognition of *T. cruzi* 80 kDa calpain by the anti-*Dm*-calpain antibody [7].

**Figure 2:**
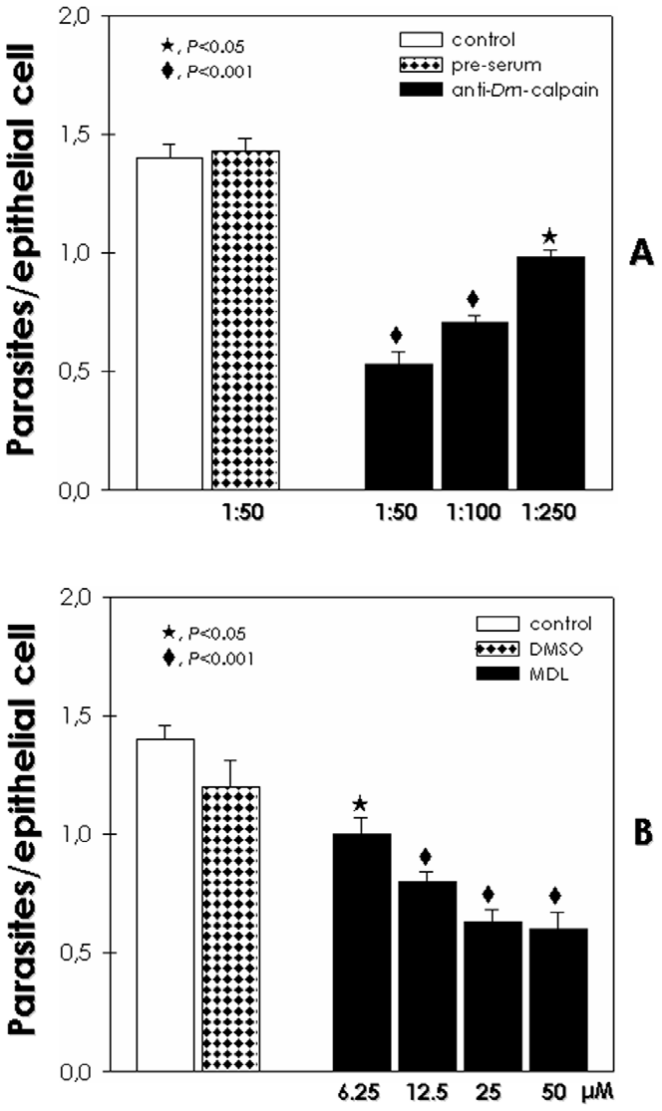
Effect of MDL28170 and anti-calpain antibody in the interaction process between *T. cruzi* and explanted guts of *Rhodnius prolixus*. (**A**) Epimastigotes (1.0×10^7^ cells) were treated for 1 h at 28°C with the anti-*Dm*-calpain (1∶50, 1∶100 and 1∶250) or the pre-immune serum (1∶50). (**B**) Epimastigotes (1.0×10^7^ cells) were treated for 30 min at 28°C with increasing concentrations of MDL28170 (6.25 to 50 µM) in 200 µl of BHI. The data from DMSO represents the concentration present in the highest dose of the drug. The viability of the parasites was not affected by the treatments used in this set of experiments. Then, parasites were washed, and incubated for 20 min at 28°C with *R. prolixus* dissected posterior midguts that were sliced open longitudinally. Subsequently, the explanted midguts were spread onto glass slides to count the number of attached parasites per epithelial cells. For each experimental group 4 insect midguts were used, and 100 epithelial cells were counted randomly. The results are shown as the mean ± standard error of the mean of three independent experiments. Symbols denote systems treated with MDL28170 or anti-calpain antibody that had a adhesion rate significantly different from the control (*P*<0.05 or *P*<0.001; Student's *t* test).

In order to assess a potential function for calpain-like molecules in *T. cruzi*, we have performed binding assays with parasites previously treated with the anti-*Dm*-calpain antibody, at concentrations that did not promote cell agglutination. The blockage of calpain molecules by the antibody led to a significant reduction in the capacity of adhesion to the insect guts by *T. cruzi* in a dose-dependent manner, the inhibition ranged from 30% to 60% as antibody concentration rose from 1∶250 to 1∶50 (Fig. 2A), similar results were obtained with the other antibodies (data not shown). On the other hand, parasites treated with the pre-immune serum at the highest concentration adhered to the guts at a rate similar to that of the control (Fig. 2A).

**Figure 3:**
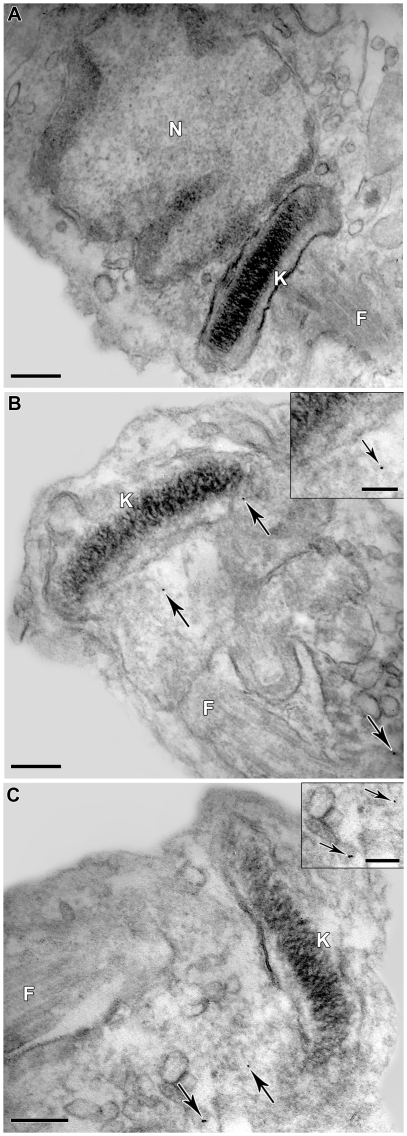
Ultrastructural immunolabelling of calpains in *T. cruzi* epimastigotes. (A) Secondary antibody control presenting no gold particles. (B,C) Parasites showing anti-calpain immunolabelling in the cytosol (arrows). In detail, it was observed in 10 nm-gold particles. Bars  =  200 nm. Inset bars  =  100 nm.

Some studies dedicated to clarify the functional mechanisms of *T. cruzi* proteins resorted to specific inhibitors as a methodological alternative [6], [7], [8], [27], [28]. In this sense, we performed binding assays with parasites previously treated with MDL28170, a potent calpain inhibitor. The compound reduced significantly the number of parasites adhered to the insect luminal midgut surface in all drug concentrations tested (6.25–50 µM). At the highest doses 25 and 50 µM, the adhesion rate was approximately 0.6 epimastigotes per midgut cell while the control was about 1.4 (Fig. 2B), which represents a reduction of 65%. DMSO at a dose equivalent to the highest concentration used to dissolve the drug did not promote any significant effect on the parasite adhesion (Fig. 2B). The effect of anti-calpain antibodies on parasite adhesion to *R. prolixus* gut together with FACS and fluorescence analyses support a surface localization of the calpains. Therefore, we performed pre-embedding assays with fixed and permeabilized parasites incubated with anti-*Dm*-Calpain at 4°C, afterwards parasites were routinely fixed and the ultrathin sections analyzed by transmission electron microscopy. The results showed labeling mainly at *T. cruzi* cytoplasm (Fig. 3), but scarce labeling in few fields was also detected at the parasite membrane (Figure S1). The parasites incubated with rabbit pre-immune serum (control) showed no labeling (Fig. 3A).

**Figure 4:**
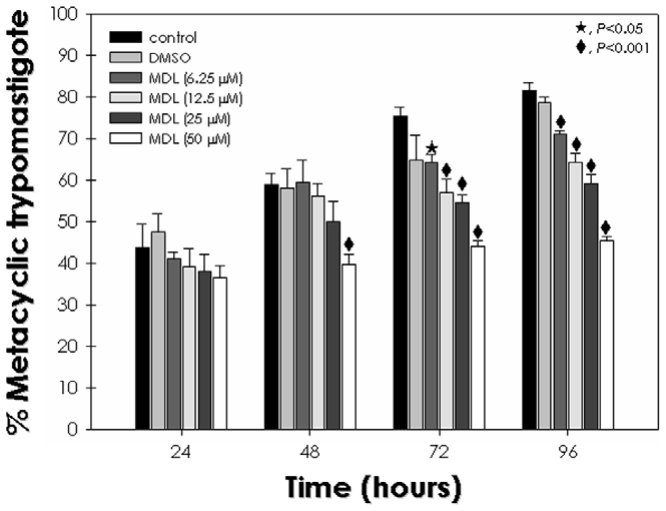
Effect of MDL28170 on *T. cruzi* metacyclogenesis *in vitro*. Epimastigotes from the stationary phase of growth (5.0×10^8^ cells) were incubated in TAU3AAG medium for 96 h to induce cellular differentiation to metacyclic trypomastigotes. Parasites were treated with increasing concentrations of MDL28170 (6.25 to 50 µM). The data from DMSO represents the concentration present in the highest dose of the drug. At each time point, culture supernatants were collected and the number of epimastigotes and metacyclic trypomastigotes was determined by cell counting in a Neubauer chamber. The results correspond to the mean of three independent experiments performed in triplicate. Symbols denote systems treated with MDL28170 that had a percentage of metacyclic trypomastigotes significantly different from the control (*P*<0.05 or *P*<0,001, Student's *t* test).

These results led us to investigate whether MDL28170 might have any effect on the metacyclogenesis process. For this purpose, we performed experiments in which late-log phase epimastigotes form the Dm28c clone were submitted to the differentiation assay, and treated or not for 4 days with MDL28170 at concentrations ranging from 6.25 to 50 µM. The Dm28c was selected instead of Y strain because this strain did not yield a satisfactory rate of differentiation to metacyclic trypomastigotes. Under this experimental condition the parasites maintained their viability, as judged by their morphology and flagellar motility in which the density of live parasites was approximately the same as the control. It should also be pointed out that MDL28170 has a trypanostatic effect on *T. cruzi*, since cells pre-treated for 72 h with the calpain inhibitor even at 70 µM resumed growth when subcultured in a drug-free fresh medium [7]. Finally, the metacyclogenesis assay revealed that the drug present a time and dose-dependent inhibition profile, since in cultures without MDL28170 the number of metacyclic trypomastigotes increased over time, whereas MDL28170 treatment inhibited the differentiation process almost to 50% in the highest drug concentration. DMSO at a dose equivalent to the highest concentration used to dissolve the drug did not interfere in the metacyclogenesis (Fig. 4). Then, we decided to investigate whether anti-calpain antibodies would prevent metacyclogenesis, all antibodies tested even at the highest concentration (1∶50) did not promote a significant effect on parasite differentiation (data not shown).

**Figure 5:**
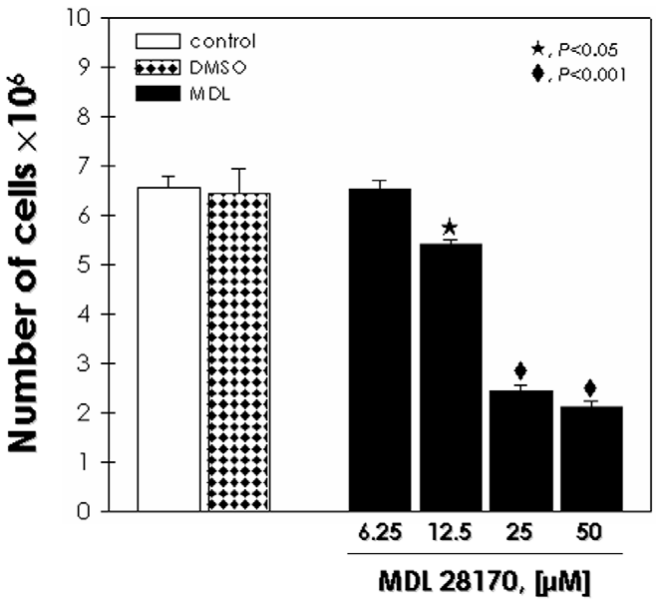
*In vitro* efficacy of MDL28170 on *T. cruzi* bloodstream trypomastigotes viability. Bloodstream trypomastigote forms obtained from Swiss mice (5.0×10^6^ cells) were treated with increasing concentrations of MDL28170 (6.25 to 50 µM) for 24 h. The data from DMSO represents the concentration present in the highest dose of the drug. Thereafter, viable parasites were counted by trypan blue exclusion and mobility. The results are expressed in viability percentage in relation to control. The results correspond to the mean of three independent experiments performed in triplicate. Symbols denote systems treated with MDL28170 that had a viability percentage significantly different from the control (*P*<0.05 or *P*<0.001; Student's *t* test).

Finally, we decided to evaluate the direct effect of the MDL28170 on bloodstream trypomastigotes viability *in vitro* after 24 h of treatment at 37°C. The inhibitor at 25 µM powerfully reduced parasite viability at about 63% in comparison to control (Fig. 5). Bloodstream trypomastigotes cultured in the presence of DMSO at a dose used to dissolve the highest drug concentration presented no significant effect (Fig. 5).

**Figure 6:**
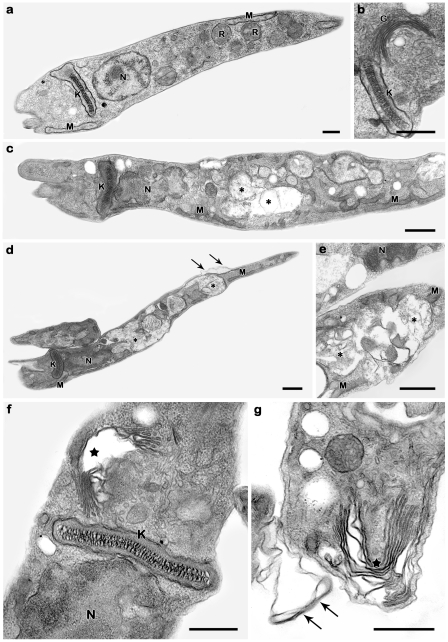
Ultrastructural effects of MDL28170 in *T. cruzi* epimastigotes. (a,b) Control parasites showing typical elongated morphology with normal kinetoplast (K), mitochondrion (M), nucleus (N) and Golgi (G). (c–g) The treatment of epimastigotes with 34 µM of the inhibitor for 72 h led to an extensive disorganization in the reservosomes (asterisks), plasma membrane alterations (arrows) as well as to the disruption of Golgi cisternae (stars). Bars  =  0.5 µm.

Given that the calpain inhibitor can reduce the growth of epimastigote forms with a trypanostatic effect [7], interfere in the parasite adhesion to the insect midgut and in the differentiation process of epimastigotes into metacyclic trypomastigotes, we decided to investigate the ultrastructural effects of MDL28170 against the epimastigote forms, by transmission electron microscopy. For this purpose, the morphology of non-treated cells (Fig. 5a–b) was compared with the ultrastructure of parasites treated for 72 h with 34 µM of MDL28170, the 50% inhibitory concentration (IC_50_) of MDL28170, as previously determined [7] (Fig. 6c–g). Results showed an important injury to the reservosomes (Fig. 6c–e), plasma membrane (Fig. 6d,g) and Golgi (Fig. 6f,g). Reservosomes and Golgi were severely affected, showing a washed-out appearance with loss of organelles' electrondensity and complete disruption of their membranes.

## Discussion

Over the past few years some studies have described the presence of calpain-related proteins in trypanosomatids. At first, a classical study employing whole genome analyses showed the presence of a large and diverse family of calpains in *T. brucei*, *L. major* and *T. cruzi* [14]. In this sense, our group became involved in the study of these molecules though the report of the expression of calpain-like molecules and the effects of the calpain inhibitor MDL28170 against the etiological agents of Chagas' disease and leishmaniasis [6], [7], [8]. In the present study, we demonstrated that MDL28170 also inhibited *T. cruzi* adhesion to the insect gut, arrested the metacyclogenesis process, reduced the bloodstream trypomastigote viability and promoted significant ultrastructural effects on reservosomes and Golgi of epimastigote forms. In addition, it was shown that anti-calpain antibodies impair parasite adhesion to the midgut surface of *R. prolixus in vitro*.

The calpain inhibitor MDL28170 impaired significantly the adhesion rate of epimastigotes to the midgut of *R. prolixus in vitro* suggesting that calpain-like molecules could play an important role in this part of the parasite life cycle. Although MDL28170 is relatively a specific calpain inhibitor, it cannot be ruled out that it may act on other *T. cruzi* cysteine peptidases to a lesser level [29]. Several classes of inhibitors, including peptidyl epoxide, aldehyde, and ketoamide inhibitors, targeting the active site have proven effective against calpains and are in the process of evaluation in animal models to treat human disease. However, a major limitation of such inhibitors is their lack of specificity. The development of a new class of calpain inhibitors that interact with domains outside of the catalytic site of calpain may provide greater specificity, which may allow to more precisely assess its function [30]. Indeed, this approach would be particularly interesting for calpain kinetoplastids since it is speculated that most calpain-like proteins do not have proteolytic activity, since the amino acid residues essential for catalytic activity are altered [14]. It has been speculated that calpains devoid of activity are involved in regulatory processes [14], [31], for instance, calpain-6 is involved in microtubules stabilization through a non-catalytic mechanism [32]. Up to date, a calpain with proteolytic activity in trypanosomatids was only described in *C. deanei,* an insect trypanosomatid. However, since its microsequencing was not performed; it is still an open question. Also, since *C. deanei* genome is not available it is not possible to search for calpain sequences with the conserved catalytic triad [16]. It is interesting to note that with few exceptions, most organisms outside the animal kingdom have only a single calpain gene, while in the Trypanosomatidae family, there is a surprising expansion of genes, which may reflect parasite plasticity to face distinct environments, such as the mammalian host and the insect vector [14], [33]. Therefore, although the results employing MDL28170 must be interpreted with caution, the anti-calpain antibodies were capable of significantly reducing the number of bound parasites to the luminal midgut surface of the insect host, which supports the possible involvement of calpains.

A first pre-requisite for calpain-like molecules to act on parasite binding to the insect midgut would be its surface location. Typically, calpains are cytosolic enzymes. Although membrane binding is not well substantiated for classical calpains, predicted transmembrane segments in phytocalpain and some ciliate calpains suggest an evolutionary link between calpain function and membranes [34]. At least two acylated calpain-like proteins in the kinetoplastids *L. major* and *T. brucei* are biochemically associated or co-localize with cellular membranes [35], [15]. Acylated proteins are often associated with the cytoplasmic face of membranes and lipid rafts, where they are implicated in signal transduction [35], [15]. Reversible externalization of intracellular proteins following reparable mechanical damage of the plasma membrane has been recently reported for cells in tissues of multicellular organisms [36]. Calpains have been described in the surface of the tegument of male adult worms, being secreted by cercarian penetration glands [37]. Whether calpains are transiently or constantly present at *T. cruzi* surface remains to be elucidated. Bioinformatics analysis gives indication of putative acylation, myristoylation and palmitoylation motifs in *T. cruzi* calpains, suggesting that they may be membrane-associated [38] (Ennes-Vidal, unpublished data). Accordingly, TcCALPx11 (XP_816697.1) partitioned in the insoluble fraction after detergent extraction, suggesting an association with membranes [39]. Additionally, calpains were identified in a proteomic analysis of detergent-solubilized membrane proteins from *T. cruzi* [38]. FACS analysis revealed a clear predomination of calpains in *T. cruzi* cytosol, since detergent permeabilization increased the fluorescence intensity [7]. The immunofluorescence analysis with non-permeabilized parasites is suggestive of calpain surface localization, and the effect of anti-*Dm-*calpain on parasite binding to the insect midgut also supports a surface location. Curiously, the pre-embedding technique did not reveal significant membrane labeling. Calpains localization in *T. cruzi* is still an open issue, and the main goal of our laboratory is to help to elucidate this question. One possible explanation would be that some isoforms, recognized by the same antibody, are abundantly expressed in the cytosol and recruits more efficiently the antibody. Indeed, some membrane labeling was detected, in scarce fields.

The metacyclogenesis process consists in the differentiation of non-infectious *T. cruzi* epimastigotes into pathogenic metacyclic trypomastigotes. During this process, epimastigotes adhere to the epithelium of the insect midgut before transforming into metacyclic trypomastigotes [2]. The adhesion is thought to be a pre-requisite for differentiation to the infective form, but less is known about how epimastigote adhesion triggers the differentiation process after the nutritional stress [40]. Our findings from *T. cruzi in vitro* metacyclogenesis showed that the calpain inhibitor impaired the differentiation process, which is not surprising since previous studies have shown that cysteine peptidases are required during the metacyclogenesis process [41], [42]. Once again, it should be taken into account that MDL28170 might be acting on other *T. cruzi* cysteine peptidases. In addition, this approach was performed with Dm28c instead of the Y strain. Distinct levels of expression of calpains were detected between Y strain and Dm28c: higher levels (twice as many) were found for Y strain in comparison to Dm28c [7]. Therefore the better performance of the Dm28c to differentiate *in vitro* does not have a direct correlation with calpain expression, at least for the isoforms detected by the anti-*Dm*-calpain. When live parasites were allowed to differentiate in the presence of the antibody, no significant effect was observed. Since we are dealing with live parasites and the incubation time required for differentiation is too long, the live parasites could be removing the proteins from the surface, since antibodies can be quickly interiorized by endocytosis and degraded [42], [43], or even, subjected to constant secretion to the surface such that bound calpains are sloughed off and replaced. Although our data do not allow to infer if calpain-like molecules are actually involved in *T. cruzi* metacyclogeneis, the transcriptome of several *T. cruzi* indicated that TcCALPx11 is up-regulated in epimastigotes under nutritional stress [39], a requirement for differentiation to metacyclic trypomastigotes [4].

Finally, the ultrastructural data demonstrated severe effects of MDL28170 in the reservosomes and Golgi, which showed a washed-out appearance with loss of organelles electrondensity and complete disruption of their membranes. These results indicate that the calpain inhibitor strongly affect *T. cruzi* epimastigote forms. Proteins and lipids accumulated in reservosomes of epimastigote forms are used as an energy source during metacyclogenesis, resulting in the disappearance of these organelles in the vertebrate stages of the parasite [44]. Besides protein degradation during metacyclogenesis, differentiating parasites must quickly synthesize new trypomastigote-specific proteins. Therefore, the effects promoted by MDL28170 in crucial organelles for protein degradation and synthesis are in accordance to metacyclogenesis inhibition. Cruzipain has been implicated as the main peptidase involved in protein degradation in reservosomes [44], [45]. Similar ultrastructural alterations were observed in epimastigotes treated with inhibitors from the major cysteine peptidase of *T. cruzi*, the cruzipain [46]. The cysteine peptidase inhibitor was capable of modifying the intracellular localization of cruzipain and induced its accumulation in peripheral dilations of Golgi cistern, these abnormalities were followed by distention of endoplasmatic reticulum and nuclear membranes [46]. It should be taken into account that MDL28170 could act nonspecifically on cruzipain. Nevertheless, a recent proteomic analysis showed the presence of calpain-like molecules in a purified fraction of reservosomes [45].

It has been almost ten years since the first calpain-like protein CAP5.5 was characterized in *T. brucei* [15]. The distribution of calpains in the ditinct life stage of *T. cruzi* is still unkown. Here, we showed that the anti-calpain antibody reacts with epimastigotes, but not bloodstream trypomastigotes, while amastiogtes were not assessed. In 2008, Giese and collaborators [39] also reported an epimastigote-specific calpain, which is one of the calpains recognized by the antibody used in the present work. Since calpains are a multigenic family, it cannot be ascertained if all calpains are indeed epimastigote-specific. Nevertheless, at least the four proteins recognized by the anti-*Dm*-calpain are epimastigote-specific, which could suggest a role in the adaptation of this life stage to the insect vector environment, as previously suggested [39]. Corroborating this view, we are demonstrating a probable involvement of calpains in insect midgut attachment, and metacyclogenesis. However, it should be reinforced that at least these four proteins have the catalytic triad altered and it is unlikely that they restrained catalytic properties. Therefore, our main goals now are a carefull analysis of calpain expression in the distinct life forms, which will help to shed some light on calpain functions.

## Supporting Information

Figure S1 **Ultrastructural immunolabelling of calpain in** ***T. cruzi*** **epimastigotes surface.** (A) The parasite presented cytosolic labeling and scarce gold-particles in the plasma and flagellar membranes (squares). (B,C) The high magnification of plasma membrane and the flagellar pocket regions evidenced the 10 nm-gold particles (arrows). A, Bar  =  200 nm. B,C, bars  =  100 nm.
(TIF)
