# Supplementary figures and images for: MDL28170, a Calpain Inhibitor, Affects Trypanosoma cruzi Metacyclogenesis, Ultrastructure and Attachment to Rhodnius prolixus Midgut
Source: PLoS One. 2011 Apr 4;6(4):e18371. doi: 10.1371/journal.pone.0018371 (PMC3070728; doi:10.1371/journal.pone.0018371)

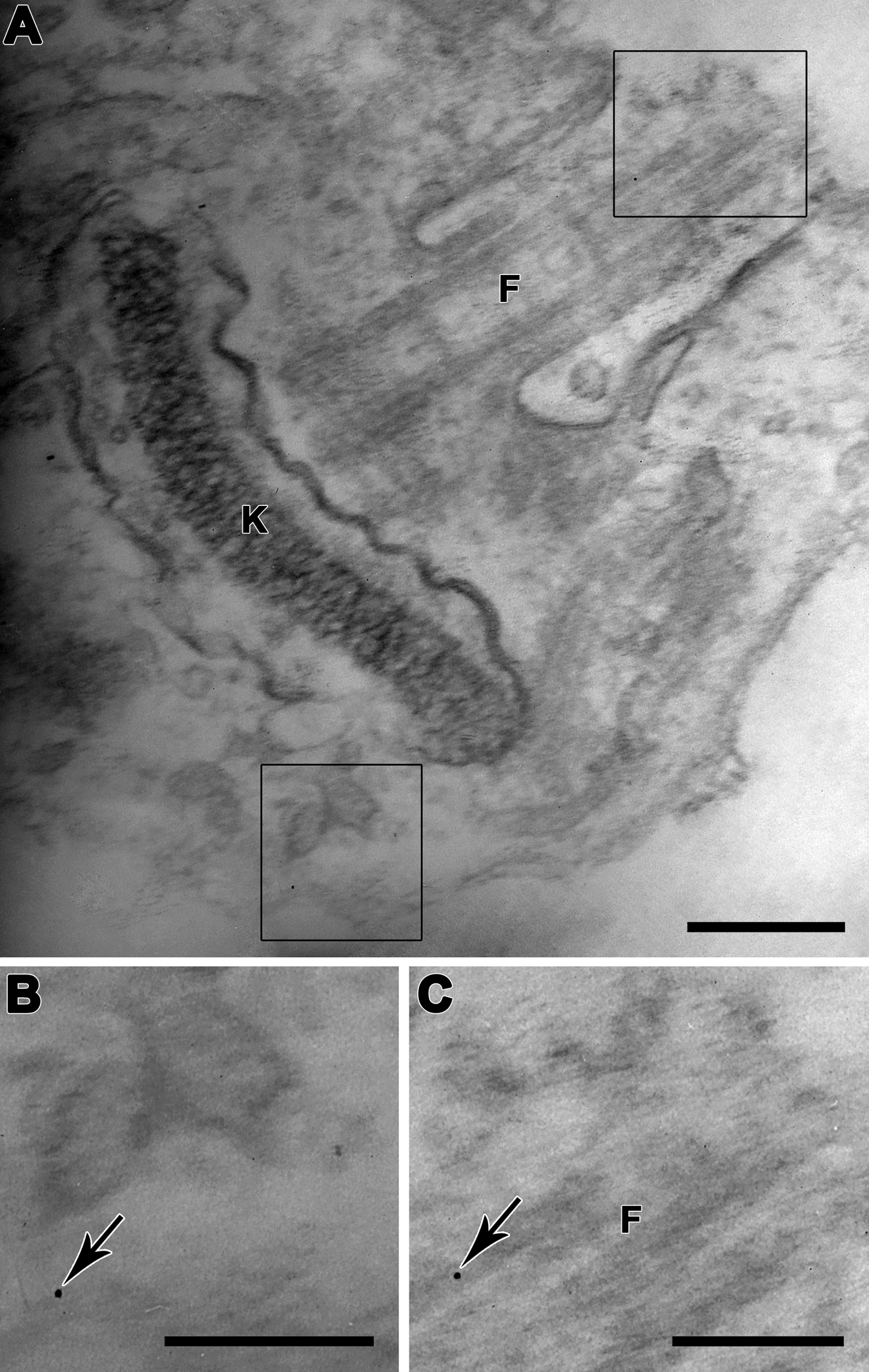

Supplement: Figure S1 — Ultrastructural immunolabelling of calpain in T. cruzi epimastigotes surface. (A) The parasite presented cytosolic labeling and scarce gold-particles in the plasma and flagellar membranes (squares). (B,C) The high magnification of plasma membrane and the flagellar pocket regions evidenced the 10 nm-gold particles (arrows). A, Bar = 200 nm. B,C, bars = 100 nm. (TIF) [file pone.0018371.s001.tif]
